# Supplementary material for: The Protective Effects of Ganoderic Acids from Ganoderma lucidum Fruiting Body on Alcoholic Liver Injury and Intestinal Microflora Disturbance in Mice with Excessive Alcohol Intake
Source: Foods. 2022 Mar 25;11(7):949. doi: 10.3390/foods11070949 (PMC8997615; doi:10.3390/foods11070949)
Supplement: Supplementary file 1 [file foods-11-00949-s001.zip › foods-1622379-supplementary.pdf]

Supplementary Materials

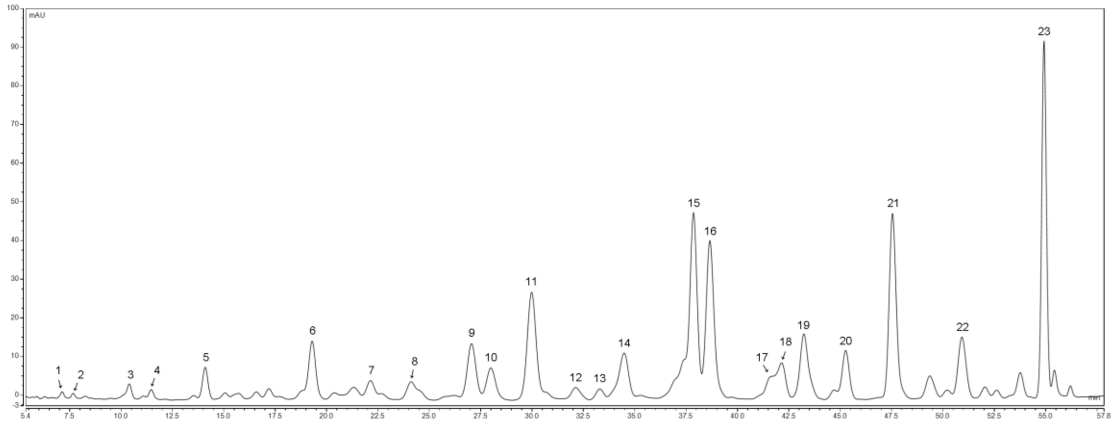

Figure S1. Phytochemical analysis of ganoderic acids (GA) performed through high performance liquid chromatography (HPLC) coupled with a QTOF electrospray ionization MS system at negative ion mode.
